# Integrating Artificial Intelligence into Orthopedic Practice: Modeling Attitudes and Intentions to Use AI

**DOI:** 10.3390/healthcare14183047

**Published:** 2026-09-17

**Authors:** Cosmin Constantin Baciu, Bogdan Mircea Măciuceanu Zărnescu, Sebastian Vâlcea, Anamaria-Cătălina Radu, Gabriela Soare

**Affiliations:** 1 Department 14 of Orthopedics and Traumatology, University of Medicine and Pharmacy “Carol Davila” Bucharest, 050474 Bucharest, Romania; constantin.baciu@umfcd.ro; 2 Clinical Emergency Hospital (SCUB), 014461 Bucharest, Romania; gabriela.soare@umfcd.ro; 3 Department of Plastic and Reconstructive Surgery, University of Medicine and Pharmacy “Carol Davila” Bucharest, 050474 Bucharest, Romania; 4 Department 10 of General Surgery, University of Medicine and Pharmacy “Carol Davila” Bucharest, 050474 Bucharest, Romania; 5 Institute of National Economy, Romanian Academy, 050711 Bucharest, Romania; anamaria_radu15@yahoo.com

**Keywords:** artificial intelligence, orthopedic practice, AI adoption, digital health, technology acceptance, clinical decision-making, healthcare technology

## Abstract

**Highlights:**

**What are the main findings?**

- A structural equation model was used to examine the factors associated with orthopedic doctors’ attitudes and intentions to adopt AI in clinical practice.
- The findings identify the key factors associated with doctors’ attitudes toward AI and their intention to adopt it in orthopedic practice.

**What are the implications of the main findings?**

- The findings provide a better understanding of the factors that may facilitate the integration of AI into orthopedic practice.
- The results may support healthcare organizations in developing strategies for the effective adoption of AI in clinical practice.

**Abstract:**

**Background/Objectives:** Artificial intelligence is increasingly used in medicine, particularly in orthopedics, to support medical image interpretation, diagnosis, clinical decision-making, and treatment selection. Its integration into clinical practice depends largely on doctors’ perceptions and attitudes toward this technology. This study aimed to identify the main factors associated with Romanian orthopedic doctors’ attitudes toward AI and their intention to use it in clinical practice. **Methods:** The study included 166 doctors specializing in Orthopedics and Traumatology in Romania. Data were collected through an online questionnaire using non-probability snowball sampling. The conceptual model examined the associations between five factors and doctors’ attitudes toward AI: perceived usefulness, perceived ease of use, trust in AI, digital competencies, and perceived advantages. The relationship between attitude and intention to use AI was also analyzed. The model was tested using structural equation modeling with WarpPLS 8.0. **Results:** All factors were positively and significantly associated with doctors’ attitudes toward AI. Perceived usefulness showed the largest association with attitude (β = 0.27), followed by digital competencies (β = 0.22). The model explained 83% of the variance in attitude. Attitude was positively associated with intention to use AI (β = 0.65), with the model explaining 43% of the variance in intention. The model showed a GoF of 0.707 and an AFVIF of 2.645. **Conclusions:** The findings may support healthcare managers in developing digitalization strategies, structured digital competency training, and clear institutional guidelines for the safe and effective use of AI in clinical practice.

## 1. Introduction

Artificial intelligence has an abundance of definitions, the most prevalent being the ability of machines to execute intricate tasks in a manner similar to human intelligence [1]. AI technologies have great potential in the orthopedic specialty due to their ability to enable more accurate diagnoses through advanced image recognition, improve surgical procedures with the aid of predictive analytics, and provide personalized treatment plans based on patient data [2]. Among the applications of artificial intelligence in orthopedic surgery are fracture diagnosis and classification, risk assessment and outcome prediction, navigation during surgical procedures, and robotic assistance in surgery. Orthopedic practice also relies on advanced technologies and materials in procedures such as joint replacement and implant-based surgery [3]. Recent technological developments in orthopedics have also supported new approaches for the treatment and monitoring of musculoskeletal conditions, including osteoarthritis and chondral lesions [4,5].

Technological advances in orthopedics also include the development of new biomaterials and 3D printing techniques, which have shown important potential in bone tissue engineering and bone regeneration [6]. AI-based solutions have become widely used in this medical specialty due to the complexities involved, such as anatomical variability and long recovery periods for patients [7]. Interest in applying artificial intelligence in orthopedic surgery has increased in recent years due to its considerable potential. Currently, AI-assisted procedures use integrated systems to support the complete surgical workflow [8].

Artificial intelligence has become a valuable tool in orthopedic surgery, providing solutions to complex situations. AI, together with other technologies such as machine learning, robotics, and computer vision, has significant potential to improve surgical precision and mitigate risks. An important subset of artificial intelligence that allows software and robotic systems to learn and acquire knowledge from various datasets is machine learning (ML). It plays a significant role in improving algorithm precision by predicting data outcomes. This is made possible by searching for and identifying patterns in input data and subsequently comparing the resulting predictions with known outcomes from stored data [9]. Machine learning can be divided into two main forms: supervised and unsupervised learning. Both are widely applied within the healthcare sector. Supervised ML focuses on developing predictive models and is extensively used in disease detection and forecasting treatment outcomes in patients. The second type, unsupervised machine learning, plays an important role in identifying hidden patterns or structures in healthcare data and is mostly used in disease subtyping [10]. In orthopedic surgery, AI-based robotics, along with navigation systems, plays a significant role in the planning and execution of operative treatments by optimizing precision, mitigating surgical risks, and reducing the time required for patient recovery. Artificial intelligence aims to meet patient needs through customized interventions [11].

### 1.1. Artificial Intelligence in Orthopedic Diagnosis, Treatment and Surgical Practice

Within orthopedic image analysis, AI algorithms enable the visualization of various structures, such as bone, cartilage, and muscle [12]. Artificial intelligence is a term that comprises a broad range of applications and refers to the use of algorithms to generate outputs in a manner similar to human thinking without requiring direct human intervention [13]. Among the applications of AI within orthopedic practice are diagnostics, including fracture recognition and tumor detection, predictive clinical models and patient outcome prediction, such as mortality rates and hospitalization, as well as postoperative patient recovery [14]. Another example of innovation in this medical field is robotic-assisted surgery, in which artificial intelligence plays a crucial role. This type of application involves a dedicated robot that is able to maximize the use of real-time data processing and actively adapt to operative requirements. The robotic system is precisely handled by specialist surgeons with the purpose of achieving improved patient outcomes, such as lower intra- and postoperative risks, increased accuracy of procedures, including prosthesis alignment, and faster recovery times [15]. Some examples of robotic-assisted systems are da Vinci and Mako [16]. Additionally, AI-based navigation and augmented reality (AR) have shown remarkable precision in spinal surgery, providing support for procedures with an increased level of complexity [17]. One of the applications of artificial intelligence that has become a central focus among researchers over the past decade is fracture recognition based on image analysis. Artificial intelligence is able to identify a variety of upper- and lower-limb fractures, such as hip and radius fractures, with very high accuracy on radiographs. An important achievement of AI in this field is its ability to detect difficult diagnoses, such as scaphoid fractures, with an efficiency comparable to that of medical professionals [18]. Another major contribution of this technology within orthopedics is tumor detection. With the help of machine learning tools, primary bone and soft tissue tumors that are not apparent on X-rays can be detected [19]. Artificial intelligence can also be used in preoperative planning for total knee arthroplasty. Nevertheless, its application is still limited by several factors, such as knee alignment and rotational variability [20]. Another important use of artificial intelligence within orthopedics is surgical training. With the aid of machine learning and computer vision, AI is able to gather data and provide relevant, customized feedback for the development of surgeons’ skills [21]. Preoperative planning represents a critical stage of orthopedic surgery by establishing safety measures for patients and influencing long-term recovery outcomes. With the aid of AI, surgeons are now able to calculate accurate trajectories to avoid sensitive structures such as blood vessels and nerves. AI tools are also able to adapt actively during procedures [22].

This technology is also used for image classification and anatomical visualization with the aid of 3D modeling tools. Detailed 3D models powered by artificial intelligence are able to reconstruct CT or MRI imaging data and recreate individual anatomy in order to train medical specialists and educate patients about surgical procedures [23]. An example of 3D modeling with AI is the Enhanced Attention Res-UNet model, which is used to optimize the precision of bone structure segmentation, extract complex anatomical characteristics, and recreate realistic models for surgical planning [24]. The U-Net architecture plays a major role in orthopedics for anatomical segmentation, as it demonstrates high efficiency in preserving fine spatial details. For instance, it can be used to map critical soft tissues, neural structures, and cartilage [25]. An example is the 3D U-Net model, which demonstrates high precision in bone structure segmentation, surgical navigation assistance, and optimized prosthesis matching [26]. Virtual reality (VR) and augmented reality (AR) AI-based tools are used to create virtual environments for simulating surgical operations prior to actual procedures. With the aid of virtual surgery, medical specialists are able to identify risks and reduce intraoperative time and complications in complex procedures, such as bone tumor resection and spinal injury surgery [27]. During surgical operations, doctors frequently have to make rapid decisions based on their clinical experience, expertise, and judgment in individual cases. This situation may lead to potential human bias in orthopedic surgery, increasing the need for intelligent decision-support systems [28]. Within orthopedic surgery, predicting prognostic outcomes is crucial for treatment decisions and the management of patient expectations. In high-risk situations, assessing mortality risk can influence treatment and care planning [29,30]. Orthopedic patients require effective management of postoperative complications, which traditionally relies on the clinical experience of medical professionals. AI integrates multimodal data and provides a customized approach for each patient [31]. In postoperative care, artificial intelligence is used to automate imaging analysis, support clinical decisions, and remotely monitor patients. In this way, AI tools can contribute to the efficiency of postoperative workflows, the early detection of complications, and the development of personalized treatment schedules [32]. Nowadays, artificial intelligence is increasingly used to communicate risks to patients. According to Dasci et al. [33], AI is able to provide accurate and accessible information, suggesting that chatbots could become an important part of preoperative planning.

### 1.2. Challenges and Future Perspectives of Artificial Intelligence in Orthopedics

Artificial intelligence in the orthopedic specialty represents a multidimensional framework that comprises different applications, ranging from surgical planning and preoperative risk prediction to postoperative treatment. In clinical settings, it is imperative that medical specialists understand whether an AI system provides a certain result or a prediction. However, these applications do not involve the same level of clinical risk. AI tools used for image analysis, screening, or decision support mainly assist doctors in their clinical work, while the final decision remains under medical supervision. AI-enhanced robotic and intraoperative systems differ because they are more directly involved in surgical procedures. Their use may therefore raise additional concerns regarding patient safety, system failure, professional responsibility, and clinical oversight. For this reason, doctors’ acceptance of AI may vary depending on the type of application and the extent to which it is involved in clinical decisions or interventions [34,35]. The quality of a decision made by an AI tool inherently relies on the input data. For instance, if the AI system fails to identify an anatomical structure, accurate implant placement cannot be achieved [36,37]. The future of AI-assisted orthopedic surgery will not involve replacing human surgeons with robots. Instead, collaborative human–robot systems are expected to be developed in order to increase surgeons’ capabilities and improve patient outcomes [38]. Shared autonomy is another important direction in the human-AI partnership. Douglas et al. [39] state that this approach allows humans to maintain ultimate authority over the procedure, while AI can handle demanding and repetitive tasks. The extensive application of AI within orthopedic surgery has created important opportunities for customized patient treatment and precision medicine, but it has also raised legal, ethical, and practical concerns. These include informed consent and patient autonomy, algorithmic bias, limited clinical acceptance, a lack of regulatory frameworks, accountability, liability, and AI system transparency [40,41]. The integration of AI tools makes informed consent and patient autonomy more complex processes, as these technologies require the collection and analysis of significant amounts of patient data. This raises concerns regarding individual rights and data privacy. Another challenge for non-specialists is fully understanding the complexity of using artificial intelligence in clinical decision-making, leaving clinicians with the task of communicating the associated risks. Patients’ ability to make informed choices can be affected by a limited understanding of the risks associated with AI-based clinical decision-making. Many patients face difficulties in evaluating potential uncertainties and risks regarding treatment suggestions based on AI decision-making [42,43].

Doctors not only have to communicate AI-based clinical advice but also its limitations and uncertainties. The “black box” nature of AI systems may create difficulties for clinicians in interpreting and trusting AI recommendations. It also makes accountability more complex, as it may be unclear who is responsible in the event of negative outcomes, thereby limiting AI adoption within surgical specialties [44,45]. The performance of artificial intelligence models depends on the variety and volume of training data in order to generate generalizable results. Therefore, a major obstacle to implementing AI in orthopedics and healthcare in general is the need to ensure patient privacy and data protection [46,47]. Another important ethical concern regarding the application of artificial intelligence within orthopedic surgery is algorithmic bias. AI models are data-dependent, and their fairness and performance rely on the quality and variety of training datasets. One example in which a lack of data diversity in algorithmic training presented a high risk of patient selection bias is the use of AI models to classify osteoporosis from chest X-rays. To mitigate this bias, multicenter and diverse datasets should be included during AI model development. In this way, algorithmic fairness can be improved, as well as sensitivity to specific variables [48,49]. When it comes to legal accountability, AI in orthopedics presents numerous challenges due to variations in approval, integration, and legal understanding at state and regional levels. To address these problems, legal adaptations and targeted policy reforms are required to promote the effective and secure integration of artificial intelligence [50,51]. Legal liability is another crucial concern regarding the adoption of AI within the healthcare sector. In the case of orthopedic procedures involving artificial intelligence or robotic assistance, responsibility becomes more complex in the event of negative outcomes or medical errors [52]. One of the barriers to implementing AI in the orthopedic specialty is the economic impact of the high costs of equipment and long-term maintenance, as well as the need for companies specialized in the repair and updating of dedicated systems [53]. Another challenge is the potential for data transmission errors due to the different data formats used in orthopedic procedures, ranging from imaging to biomechanical sensors. Furthermore, the lack of system-level interoperability plays an important role in orthopedic surgery, as variations between devices may cause delays in real-time data synchronization and prevent smooth integration into orthopedic workflows [54]. A major impediment to implementing AI-assisted orthopedic surgery is the human factor. Orthopedic surgeons must trust AI systems in order to use them efficiently. They also need to understand the logic behind AI-generated recommendations. Without trust and clarity, surgeons may be reluctant to integrate AI systems into their clinical workflows [55].

High technological costs and acceptance among medical professionals remain two major obstacles to implementing AI in healthcare. A large number of medical institutions lack the financial capacity to acquire and maintain AI-based surgical equipment [56]. When dealing with complex cases, medical professionals may prefer to rely on their clinical experience rather than on recommendations based on AI decision-making [57]. The quality of the data used to train AI algorithms plays a crucial role in determining the accuracy of their outputs. If the training dataset is biased, the outcomes generated by AI systems may also be biased. For example, if data from a particular demographic group are predominantly used in algorithm development, predictions may be less accurate for other groups [58]. The widespread adoption of AI within orthopedic surgery will depend on the development of appropriate legal and regulatory frameworks. Traditional regulations may no longer be sufficient to address the autonomous and dynamic behavior of AI-based systems. Future regulations will have to balance innovation and safety in order to support the use of AI tools in orthopedics while also protecting patient rights [59,60]. The current state of AI requires a coordinated effort to successfully move from the conceptual stage toward broader application in clinical practice.

In conclusion, artificial intelligence is intended to complement the clinical decision-making process rather than serve as a stand-alone alternative. This technology has the potential to reduce the workload of medical specialists while improving patient outcomes. In the future, hybrid models combining AI, particularly machine learning techniques, with medical expertise are likely to be increasingly implemented. These tools will not only be intelligent but also applicable to real clinical scenarios.

### 1.3. Research Hypotheses

Considering the findings of previous studies in the literature, it was deemed necessary to conduct quantitative research to identify the main factors that may be associated with orthopedists’ intention to adopt artificial intelligence (AI) in orthopedic practice. For this purpose, a conceptual model was developed based on six hypotheses formulated according to relationships identified and validated in previous research. The main variables included in the proposed conceptual model are perceived usefulness, perceived ease of use, trust in AI, digital competencies, perceived advantages, and attitude toward AI use. The model aims to examine how these factors are associated with orthopedists’ attitudes toward AI and, subsequently, with their intention to adopt this technology in orthopedic practice. The research findings may have both theoretical and practical implications. From a theoretical perspective, the study may contribute to a better understanding of the factors that explain AI adoption among orthopedic doctors. From a practical perspective, the findings may highlight the factors that facilitate or support the use of this technology in clinical practice. This information may also be useful to healthcare facility managers in developing strategies to facilitate the integration of AI into medical practice and support doctors in the process of adopting new technologies. From a methodological perspective, the relationships between the variables were integrated into a conceptual model, which was subsequently tested using WarpPLS 8.0. Based on the theoretical foundation and the findings of previous studies, the following hypotheses were formulated:

**H1.**  *The perceived usefulness of AI is positively associated with orthopedists’ attitudes toward the use of AI in orthopedic practice.*

Perceived usefulness has previously been studied within the Technology Acceptance Model (TAM). It reflects the extent to which a person believes that using a technology can improve their professional performance [61]. In the medical field, this perception is important because the adoption of a new technology largely depends on the benefits that doctors believe it can bring to clinical practice. Previous studies in the literature [62] have demonstrated the significant role of perceived usefulness in the AI acceptance process. Moreover, other research [63] has shown that perceived usefulness is an important component of how doctors evaluate and intend to use these emerging technologies. More recently, empirical research among doctors has confirmed that perceived usefulness remains an important determinant of AI acceptance and behavioral intention in clinical practice [62]. In orthopedics, perceiving AI as a tool capable of improving professional practice may therefore be associated with a more favorable attitude toward its use.

**H2.**  *The perceived ease of use of AI is positively associated with orthopedists’ attitudes toward the use of AI in orthopedic practice.*

Perceived ease of use reflects how easy a technology is perceived to be by its users [61]. This aspect has also been studied in relation to the adoption of medical technologies. Chien et al. [63] found that ease of use was relevant to doctors’ acceptance of clinical decision support systems. Cicin and Çetin Gürkan [62] reported a similar relationship in the case of AI acceptance.

**H3.**  *Orthopedists’ trust in AI is positively associated with their attitudes toward the use of AI in orthopedic practice.*

Trust is considered a very important factor in the acceptance of AI in the medical field. It is particularly relevant because the outputs generated by AI-based systems may be used to support decisions with direct implications for patients. Asan et al. [64] highlighted the central role of clinician trust in interactions with AI-based systems. Moreover, they showed that a lack of trust may represent an important barrier to technology use. Other authors [65] have shown that there are direct and positive relationships between trust in technology and attitudes toward AI use among healthcare professionals. Recent empirical evidence among doctors further indicates that trust plays a central role in AI acceptance, linking doctors’ perceptions of AI with their behavioral intention to use these technologies [66]. Thus, a higher level of trust in the results and recommendations generated by AI may be associated with a more favorable attitude toward its use in orthopedic practice.

**H4.**  *Orthopedists’ digital competencies are positively associated with their attitudes toward the use of AI in orthopedic practice.*

The use of AI-based technologies requires competencies that enable healthcare professionals to properly understand and evaluate digital tools. A higher level of digital competencies may reduce the perceived difficulties associated with interacting with new technologies. Authors who have studied this field [65] have shown that there is a direct relationship between knowledge and use of eHealth and attitudes toward AI use. Recent empirical evidence also supports this relationship, showing that higher AI literacy and digital health competence are associated with more positive attitudes toward AI among healthcare professionals [67]. These findings suggest that the ability to use and understand digital technologies is relevant to how healthcare professionals perceive AI.

**H5.**  *The perceived advantages of AI use are positively associated with orthopedists’ attitudes toward the use of AI in orthopedic practice.*

When adopting a new technology, users may consider whether it offers improvements over the methods they already use. These perceived improvements represent an important aspect of technology adoption [68]. Previous research on the use of AI in the medical field has identified relative advantage as an important factor in the adoption process [69,70]. Another study conducted in this field [71] showed that perceived advantages influence doctors’ attitudes toward these technologies, with relative advantage being considered one of the strongest predictors among the factors analyzed. This relationship has also been analyzed in surgical settings, where relative advantage was identified as a major predictor of attitudes toward AI-enhanced surgical systems [71].

**H6.**  *A favorable attitude toward the use of AI in orthopedic practice is positively associated with orthopedists’ intention to adopt AI in their clinical practice.*

**Figure 1:**
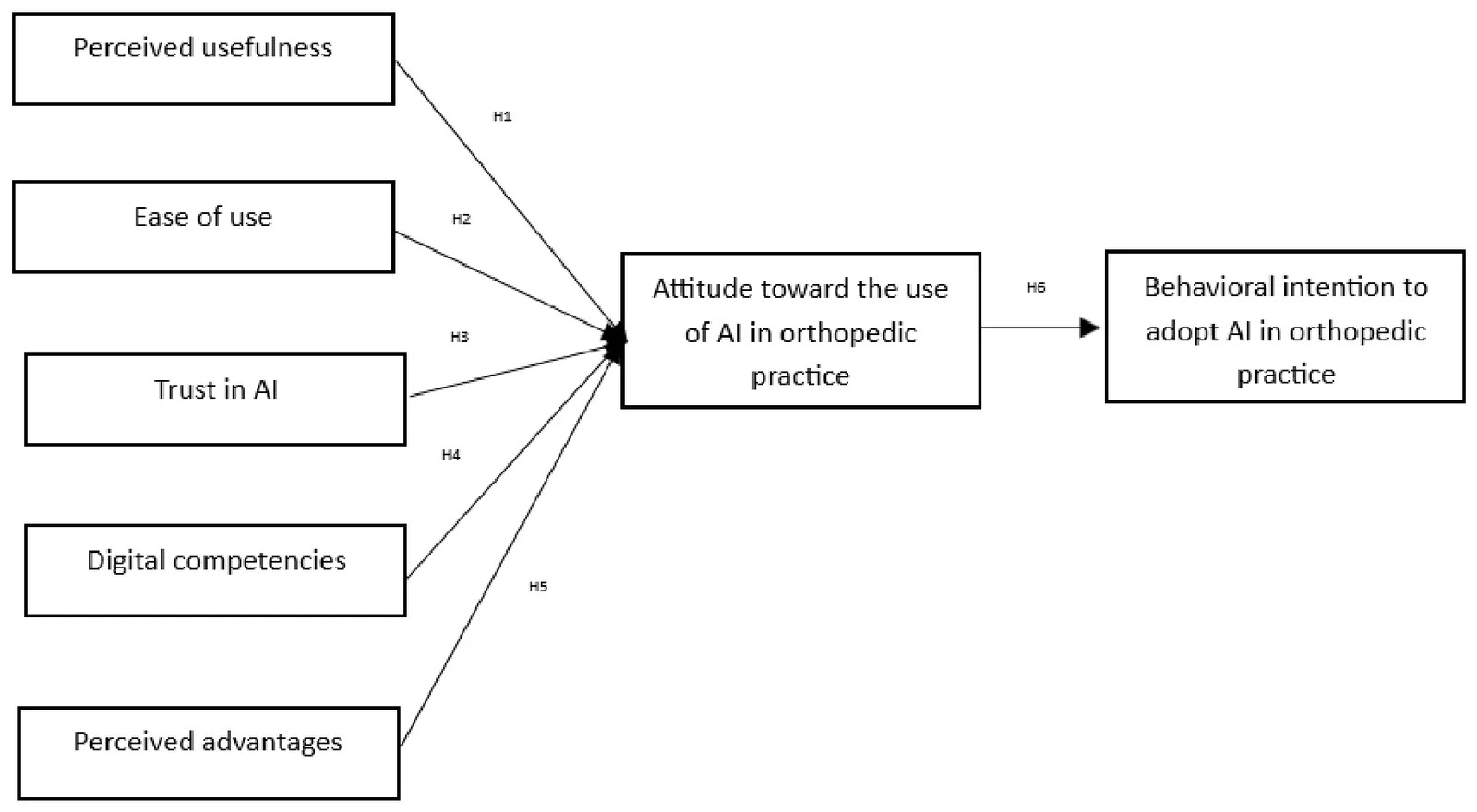
Proposed conceptual model of artificial intelligence adoption in orthopedic practice.

Doctors’ attitudes toward the use of new emerging technologies are considered to be among the most important factors in explaining their behavioral intention [72]. Wagner et al. [73] showed that a favorable attitude toward AI is associated with a higher intention to use it in medical practice. Moreover, other studies conducted among doctors [62] confirmed the importance of attitude in explaining AI acceptance. Based on the hypotheses presented above, the conceptual model illustrated in Figure 1 was developed. Testing this model allows the evaluation of the relationships between the factors considered relevant to the formation of attitudes toward AI and orthopedists’ intention to adopt this technology in clinical practice. To test the proposed hypotheses, a cross-sectional survey was conducted in Romania in 2026. The study included 166 doctors specializing in Orthopedics and Traumatology. Participants had different levels of professional experience and worked in both public and private healthcare settings. Data were collected through an online questionnaire using snowball sampling. The proposed relationships were then tested using PLS-SEM.

## 2. Materials and Methods

The study conducted as part of this research was carried out in 2026. The results obtained provide a current perspective on the main factors associated with orthopedic doctors’ attitudes toward the use of artificial intelligence (AI), as well as their intention to use this technology in the future in their professional practice. On the one hand, the study aims to highlight doctors’ perceptions of new emerging technologies and, on the other hand, to identify the factors that may be associated with their attitudes and, subsequently, their intention to adopt AI in orthopedic practice. The research provides added value for both orthopedic practitioners and the scientific community. Identifying the factors associated with doctors’ attitudes, as well as examining the relationship between these attitudes and their intention to use AI, may provide a better understanding of the process of adopting this technology in orthopedic practice. Moreover, the results may serve as a starting point for developing strategies to facilitate the integration of AI-based technologies into clinical practice. To conduct the research, a series of previously published studies on similar topics were analyzed. The analysis of secondary data contributed to a better understanding of the field and to the identification of the main factors that could influence doctors’ behavior regarding the use of AI. Based on the literature, the variables included in the conceptual model were established and the research hypotheses were formulated. Primary data were collected through a questionnaire distributed online to doctors working in the field of orthopedics. The research method used was a survey, and snowball sampling was chosen to select the participants. The final sample consisted of 166 orthopedic doctors at different stages of their professional careers, ranging from resident doctors to doctors with higher professional grades. The adequacy of the sample size was also considered in relation to the structural model. The most complex endogenous construct in the model had five predictors. Assuming a medium effect size (f^2^ = 0.15), a significance level of 0.05, and a statistical power of 0.80, a minimum sample of approximately 92 participants is required for a model with five predictors [74]. The final sample of 166 doctors therefore exceeded this minimum requirement. Prior to its distribution to the final sample, the questionnaire was pretested on a group of 15 respondents. The purpose was to identify potential wording issues and assess the clarity of the questions. Minor adjustments were subsequently made to several items. The pretest was intended to assess wording and clarity rather than to establish formal content validity. These changes did not affect the overall structure of the questionnaire. The study was conducted in accordance with the relevant ethical principles for research involving human participants. The study protocol received the necessary ethical approval before the research began. Regarding the questionnaire distribution process, orthopedic doctors working in both public and private hospitals across Romania were invited to participate in the study. Initial direct recruitment was more concentrated in Bucharest and the counties of Cluj, Timiș, Bihor, Constanța, Brașov, and Iași, where a larger number of potential participants were contacted, while invitations were also distributed to doctors working in other areas of the country. The survey invitation and questionnaire link were distributed directly to doctors known to the research team through e-mail and other digital communication channels. These doctors were asked to further distribute the questionnaire to eligible orthopedic colleagues within their professional networks. Because recruitment proceeded through successive snowball referrals and the survey link could be redistributed beyond the initial contacts, the total number of doctors who received the invitation could not be reliably determined. In addition, the exact number of initial direct invitations was not systematically recorded during data collection. Consequently, a conventional response rate could not be calculated. A total of 166 eligible and complete responses were included in the final analysis. To reduce the risk of duplicate responses, the online survey platform was configured to allow only one submission from the same identified IP address.

### 2.1. Questionnaire Design

To collect the data required for the research, a questionnaire was developed and subsequently distributed to doctors working in the specialty of Orthopedics and Traumatology. The questionnaire was designed to allow both the identification of respondents’ characteristics and the assessment of factors potentially associated with the adoption of artificial intelligence (AI) in orthopedic practice. The research instrument consisted of 28 questions. These were grouped into five sections. The first question in the questionnaire (Q1) was a screening question and was intended to select only doctors working in the field of Orthopedics and Traumatology. Its purpose was to allow only individuals belonging to the population under study to participate in the research.

In terms of its structure, the questionnaire included the following sections:

- Screening Question—Selection of the target population
  ∘ Q1—identification of respondents working professionally in the specialty of Orthopedics and Traumatology in Romania.
- Section A—Profile of orthopedic doctors
  ∘ Q2–Q7—questions aimed at creating the professional profile of the respondents. This section examined professional grade, experience in medicine, the type of healthcare facility in which they work, the area in which the healthcare facility is located, the main field of activity within orthopedics and traumatology, and the frequency of digital technology use.
- Section B—Use of Artificial Intelligence in professional practice
  ∘ Q8–Q14—This section included questions aimed at assessing respondents’ experience regarding the use of AI and digital technologies in medical practice. The questions sought to identify the use of AI-based tools or applications, as well as the areas in which these tools are used. Moreover, the main perceived barriers to AI use, participation in courses or training programs, level of knowledge about AI, available digital infrastructure, and frequency of using digital tools to support clinical decision-making were analyzed.
- Section C—Factors influencing the adoption of Artificial Intelligence
  ∘ Q15–Q21—This section analyzed the perceived usefulness of AI, perceived ease of use, trust in AI, competencies for using digital technologies, perceived advantages of AI use, doctors’ attitudes toward the use of AI in orthopedic practice, and their intention to use this technology in clinical practice.
- Section D—Perception of Artificial Intelligence in general
  ∘ Q22–Q24—The questions in this section aimed to identify doctors’ perceptions of the application of AI in orthopedic practice. The stage of orthopedic practice in which AI could generate the most important benefits, the main risk associated with its use, and the measure considered most important for facilitating AI adoption among orthopedic doctors were analyzed.
- Section E—Demographic profile of orthopedic doctors
  ∘ Q25–Q28—This section included the questions required to establish the demographic profile of the respondents. Thus, the questions addressed sex, age, monthly net income, and area of residence.

A seven-point Likert scale (1 = Strongly Disagree to 7 = Strongly Agree) was used to measure the variables included in the conceptual model. This scale was used for questions Q15–Q21. The remaining questions were formulated using nominal and ordinal scales, depending on their respective objectives. The questions included in this questionnaire were intended to establish the respondents’ professional and demographic profiles, their experience in using digital technologies and AI, as well as their opinions regarding the benefits, risks, and barriers associated with the use of AI in orthopedic practice. The variables included in the proposed conceptual model were operationalized through items. These were included in Section C of the questionnaire. The items used to operationalize the constructs were developed based on the theoretical concepts identified in the literature. They were adapted to the context of AI use in orthopedic practice. For each construct, the questionnaire included items covering its main dimensions, as described below. The questionnaire was developed and administered in Romanian. Therefore, no translation or back-translation procedure was required.

The first variable analyzed in the conceptual model was the perceived usefulness of AI. It was measured using five items. The items aimed to identify the extent to which doctors believe that the use of AI can improve the quality of their professional practice, increase the efficiency of clinical activities, support clinical decision-making, and contribute to improving professional performance. Moreover, respondents’ perceptions of the actual benefits that AI could bring to clinical practice were assessed. The second variable included in the model was the perceived ease of use of AI. It was also measured using five items. The questions aimed to assess the extent to which doctors believe that they could easily learn to use AI-based tools. Moreover, they sought to determine whether interaction with these tools would be easy to understand. At the same time, the perceived effort required to use these technologies, the speed of becoming familiar with them, and the ease of integrating them into professional practice were analyzed. Another variable analyzed in the model was trust in artificial intelligence. This construct was based on five items. The items aimed to determine doctors’ level of trust in the information provided by AI-based tools, the recommendations generated by these tools, and the reliability of the results provided. Moreover, the doctors who participated in this research were asked whether they would take into consideration information provided by an AI-based system in the clinical decision-making process and whether they would consider such a tool a reliable source of support for orthopedic doctors. Doctors’ competencies in using digital technologies and AI represented the fourth variable analyzed in the model. This variable was measured using five items. Doctors’ perceived digital competencies, confidence in their own ability to learn to use new technologies, and ability to understand the general functioning of digital tools were assessed. The items considered in the model also addressed their ability to adapt to the introduction of new technologies and their ability to properly use an AI-based tool after appropriate training. Another variable analyzed in the proposed model was the perceived advantages of using AI in orthopedic practice. This construct was measured using five items. These items addressed doctors’ perceptions of the advantages of AI compared with traditional methods. This variable examined the efficiency of clinical activities, improvements in the analysis of information required for decision-making, and the additional opportunities provided by AI. Overall, the aim was to identify the extent to which doctors consider the use of AI an advantage for orthopedic practice compared with the exclusive use of current methods. Another variable considered in the proposed conceptual model was orthopedic doctors’ attitude toward the use of AI. For this question, respondents were asked to indicate their attitude toward the use of artificial intelligence in orthopedic practice. The final variable analyzed in the model was the intention to use AI. It was also measured using a single item. Doctors were asked whether they intended to use AI-based technologies in orthopedic practice when these technologies become available and appropriate for clinical practice. As we have mentioned, attitudes toward AI use and intention to use AI were each assessed using a single item, providing a direct overall measure of these two constructs. Unlike the other constructs included in the model, they were not measured using multiple indicators. This should be considered when interpreting the results, as single-item measures may not capture the full complexity of attitude and behavioral intention. The use of all the variables presented above in the proposed conceptual model made it possible to examine the factors associated with orthopedic doctors’ attitudes toward AI. In addition, it made it possible to examine their intention to use this technology in clinical practice. The additional questions included in the questionnaire were intended to identify their experience with artificial intelligence, the barriers, benefits, and risks associated with its use, as well as the measures required to facilitate its adoption in healthcare facilities across the country.

### 2.2. Statistical Analysis and Tests

The suitability of the data for factor analysis was assessed using the Kaiser–Meyer–Olkin (KMO) measure and Bartlett’s test of sphericity. The reliability of the multi-item constructs was assessed using Cronbach’s alpha (α) and Composite Reliability (CR). Values of 0.70 or higher were considered acceptable. Convergent validity was assessed using the Average Variance Extracted (AVE). An AVE value of at least 0.50 was considered acceptable. Discriminant validity was assessed using the Fornell–Larcker criterion. The square root of the AVE for each multi-item construct was compared with its correlations with the other constructs. Discriminant validity was considered acceptable when the square root of the AVE was higher than the corresponding correlations. These analyses were performed for perceived usefulness, perceived ease of use, trust in AI, digital competencies, and perceived advantages. Attitude toward AI use and intention to use AI were measured using single items. Therefore, internal consistency measures were not calculated for these two variables. The research hypotheses were tested using Partial Least Squares Structural Equation Modeling (PLS-SEM). The analysis was performed using WarpPLS 8.0. Path coefficients and their statistical significance were examined to test the relationships included in the conceptual model. The explanatory power of the model was assessed using the coefficients of determination (R^2^) for the endogenous variables. Several indices were also used to assess the quality of the structural model. These included the Average Path Coefficient (APC), Average R-squared (ARS), and Average Adjusted R-squared (AARS). The Average Full Collinearity VIF (AFVIF) was used to assess potential collinearity. A value of 3.3 or lower was considered ideal. The overall goodness of fit was assessed using the Tenenhaus GoF index. A GoF value of 0.36 or higher was considered indicative of a large fit. The Simpson’s Paradox Ratio (SPR), R-squared Contribution Ratio (RSCR), Statistical Suppression Ratio (SSR), and Nonlinear Bivariate Causality Direction Ratio (NLBCDR) were also examined as additional model quality indices.

## 3. Results

### 3.1. Respondent Profile

A total of 166 doctors participated in this study. All respondents confirmed that they were professionally active in the specialty of Orthopedics and Traumatology. In terms of professional grade, 31.3% of the participants reported that they were resident doctors, 38% were specialist doctors, and 30.7% were senior doctors. Thus, a relatively balanced distribution of respondents according to professional grade can be observed. Regarding experience in medicine, including the residency period, 19.3% of the respondents had less than 5 years of experience, 27.7% between 5 and 10 years, 19.3% between 11 and 15 years, 15.1% between 16 and 20 years, while 18.7% had been practicing medicine for more than 20 years. Regarding the type of healthcare facility in which respondents mainly work, 42.2% work in a public hospital, while 15.7% work in a private hospital or clinic. A significant percentage, 40.4%, work in both the public and private healthcare systems, while 1.8% work in other types of healthcare facilities. In terms of the location of their main healthcare facility, 96.4% of doctors work in urban areas and only 3.6% in rural areas. The analysis of the main field of activity showed that 28.9% of respondents work in general orthopedics, making this the most represented category. Traumatology was reported by 16.9% of doctors, arthroplasty/reconstructive surgery by 12.7%, and knee surgery and/or arthroscopy by 12%. Shoulder and upper limb surgery represents the main field of activity for 9% of respondents, pediatric orthopedics for 7.8%, foot and ankle surgery for 6.6%, and spine surgery for 6%. Analyzing the respondents’ profile in terms of demographic variables, 79.5% of the research participants were men and 20.5% were women. Regarding their age, 10.2% were under 30 years old, 32.5% were between 30 and 39 years old, 27.7% between 40 and 49 years old, 21.1% between 50 and 59 years old, and 8.4% were 60 years old or older. Regarding monthly net income, 8.4% of respondents reported incomes below RON 7500, while 14.5% reported incomes between RON 7500 and 9999. A percentage of 27.7% reported a monthly net income between RON 10,000 and 14,999, while 24.7% reported between RON 15,000 and 19,999. Incomes between RON 20,000 and 24,999 were reported by 13.3% of respondents, while 11.4% reported incomes of at least RON 25,000. In terms of respondents’ area of residence, 97% of the participating doctors live in urban areas, while only 3% live in rural areas.

### 3.2. Use of AI in Professional Practice

The use of digital technologies in professional practice is high within the analyzed sample. None of the respondents reported using such technologies. Only 1.2% use them very rarely, 2.4% rarely, and 7.2% occasionally. In contrast, 20.5% use them frequently, 30.1% very frequently, and 38.6% daily. The mean score obtained was 5.92. Regarding the actual use of AI-based tools or applications in medical practice, 12% of respondents reported using them frequently, while 28.3% use them occasionally, and 22.9% have tested such tools without using them regularly. A total of 31.3% have not used such tools but are familiar with them, while only 5.4% reported that they have not used them and are not familiar with them. The results also showed that, although the current use of AI is not yet widespread, most doctors included in the study have at least some degree of familiarity with these technologies. Regarding the area in which doctors use or would primarily use AI, 33.1% indicated the analysis and interpretation of medical images. This was followed by diagnostic support (20.5%) and treatment planning (13.9%). Medical research and education were indicated by 10.8% of doctors, prognosis and risk assessment by 9%, administrative activities and medical documentation by 7.2%, and patient monitoring and follow-up by 5.4%. Regarding the barriers that may limit the use of AI in orthopedic practice, lack of knowledge and training in AI was the most frequently mentioned, by 23.5% of respondents. Lack of trust in AI-generated results was mentioned by 19.3%, while costs associated with implementation were mentioned by 16.3%. A total of 13.9% mentioned the lack of adequate technological infrastructure, while 12% indicated the difficulty of integrating AI into the clinical workflow. Legal issues and professional liability were considered the main barrier by 8.4% of respondents, while the protection and confidentiality of patient data were indicated by 6.6%. Regarding AI training, only 32.5% of respondents had participated, up to the time of the study, in courses, conferences, or training programs concerning its use in medicine, while 67.5% had not participated in such activities. Thus, 3.6% of respondents rated their level of knowledge as very low, 15.7% reported a low level, 44.6% a moderate level, 27.7% a high level, and 8.4% a very high level. Regarding the digital infrastructure available in healthcare facilities, 6% of respondents believed that it enables the implementation of AI-based technologies to a very small extent, while 18.1% indicated to a small extent. The largest category, representing 38.6% of participants, expressed a neutral opinion. On the other hand, 25.3% considered that the necessary infrastructure is available to a large extent, while 12% considered it available to a very large extent. The mean score of 3.19 indicated a moderate, slightly favorable perception of the digital infrastructure available for AI implementation. The use of digital tools to support clinical decision-making is also relatively frequent. Only 3.6% of respondents never use them, 4.2% use them very rarely, and 10.8% rarely. A total of 25.3% use them occasionally, 30.1% frequently, and 25.9% very frequently.

Regarding the stage of orthopedic practice in which AI could provide the most important benefits, 30.1% of doctors indicated diagnosis, which represented the most frequent response. Preoperative planning was mentioned by 21.1% of respondents, while treatment selection and personalization were mentioned by 18.1%. A total of 11.4% of respondents mentioned the performance of surgical procedures and postoperative monitoring. Prevention and early detection of conditions were mentioned by 6% of those who participated in the study, while rehabilitation and long-term monitoring were mentioned by 1.8%. These results showed that doctors perceive the most important benefits of AI particularly in the diagnostic and treatment preparation stages. Regarding the risks associated with AI use, 28.3% of respondents mentioned incorrect results or recommendations. The presence of errors or biases in algorithms was indicated by 24.1% of respondents, while excessive doctor dependence on technology was mentioned by 22.9%. Issues concerning the confidentiality and security of patient data were mentioned by 14.5% of participants, while 7.8% considered the main risk to be a reduction in direct doctor–patient interaction. Only 2.4% of respondents believed that the use of AI does not involve significant risks. Regarding the measures that could facilitate AI adoption among orthopedic doctors, 21.7% of respondents indicated the availability of strong scientific evidence regarding the effectiveness and safety of these tools. The organization of courses and training programs dedicated to AI use was mentioned by 19.9%, while the development of clear professional guidelines was mentioned by 14.5%. A total of 12.7% of study participants mentioned improving the digital infrastructure of healthcare facilities, 10.8% mentioned clarifying legal liability, while 10.2% indicated the integration of AI-based tools into the information systems already used in hospitals. Ensuring the protection and security of patient data was mentioned by 6%, while financial support for the implementation of these technologies was mentioned by 4.2% of respondents.

### 3.3. Reliability and Factorial Adequacy

The reliability and convergent validity of the constructs were assessed using Cronbach’s alpha, composite reliability, and Average Variance Extracted (AVE) (Table 1). Cronbach’s alpha values ranged from 0.877 to 0.913, while composite reliability values ranged from 0.911 to 0.935. All values were above the recommended threshold of 0.70, indicating good internal consistency. The AVE values ranged from 0.671 to 0.743 and were above the recommended threshold of 0.50. These results support the reliability and convergent validity of the multi-item constructs included in the model. ATT and INT were measured using single items and were therefore not interpreted in terms of internal consistency in the same way as the multi-item constructs.

The indicator loadings and cross-loadings were examined to further assess the measurement model. Each indicator loaded highest on its corresponding construct. The primary loadings were higher than the loadings on the other constructs. This pattern supports the discriminant validity of the measurement model (Table 2).

The inner VIF values for the predictors of ATT ranged from 1.38 to 2.83, while the VIF for ATT as the sole predictor of INT was 1.00. All values were below the recommended threshold of 3.3, indicating that collinearity was not a concern in the structural model (Table 3).

Discriminant validity was assessed using the Fornell–Larcker criterion. The square root of the AVE for each construct was compared with the correlations between constructs. As shown in Table 4, the values obtained for the multi-item constructs were higher than their correlations with the other constructs. The highest correlations were found between perceived usefulness and perceived advantages, perceived usefulness and attitude, and perceived advantages and attitude. These values remained below the corresponding square roots of the AVE. Therefore, the results support the discriminant validity of the multi-item constructs. ATT and INT were measured using single items. For this reason, their diagonal values of 1.000 should not be interpreted in the same way as those of the multi-item constructs.

The suitability of the data for factor analysis was assessed using the Kaiser–Meyer–Olkin (KMO) measure and Bartlett’s test of sphericity. The KMO value was 0.823, indicating good suitability of the data for factor analysis. Bartlett’s test was statistically significant, χ^2^(300) = 5305.990, *p* < 0.001, confirming the presence of sufficient correlations among the analyzed items (Table 5).

### 3.4. Testing of the Structural Model

WarpPLS 8.0 software was used to test the proposed conceptual model. The purpose of the analysis was to identify the relationships between the variables included in the model. Thus, the associations between perceived usefulness, perceived ease of use, trust in artificial intelligence, digital competencies, perceived advantages, and orthopedic doctors’ attitudes toward the use of AI were analyzed. Furthermore, the relationship between attitudes toward AI and their intention to use this technology in medical practice was analyzed. Six hypotheses were formulated and tested within the conceptual model. The results (Table 6) showed that all the relationships analyzed were positive and statistically significant. Thus, all six hypotheses were supported by the research results. The first hypothesis analyzed the relationship between perceived usefulness and attitude toward AI use. The results showed a positive and statistically significant relationship (β = 0.27, *p* < 0.01). Therefore, H1 was supported. This result shows that a higher perceived usefulness of AI is associated with a more favorable attitude toward the use of this technology. The second hypothesis examined the association between perceived ease of use and attitude. The identified relationship was positive and statistically significant (β = 0.18, *p* < 0.01). Thus, H2 was supported. In the case of the third hypothesis, the results showed a positive relationship between trust in AI and doctors’ attitudes (β = 0.18, *p* < 0.01). The relationship was statistically significant. Therefore, H3 was supported. The fourth hypothesis analyzed the relationship between digital competencies and attitude toward AI. The results showed a positive and statistically significant relationship (β = 0.22, *p* < 0.01), thus supporting H4. In the case of perceived advantages, a positive and statistically significant relationship with attitude toward AI was also identified (β = 0.21, *p* < 0.01), and H5 was therefore supported. Among the five factors analyzed, perceived usefulness showed the strongest relationship with attitude (β = 0.27). It was followed by digital competencies (β = 0.22) and perceived advantages (β = 0.21). The final hypothesis analyzed the relationship between doctors’ attitudes toward AI and their intention to adopt the technology in clinical practice. The results showed a strong positive relationship (β = 0.65, *p* < 0.01). Thus, H6 was supported. This was the strongest relationship identified within the model. The result shows that a more favorable attitude toward AI is associated with a greater intention to use it in orthopedic practice.

The analysis of the coefficients of determination (R^2^) shows the model’s ability to explain the variation in the dependent variables. In the case of attitude toward AI, the R^2^ value was 0.83. Thus, 83% of the variation in attitude is explained by the five variables included in the model. In the case of the intention to adopt AI, the R^2^ value was 0.43. Therefore, 43% of the variation in intention is explained by doctors’ attitudes toward the use of AI (Table 7).

In addition to the indicators mentioned above, the model quality indicators were also examined. The APC value was 0.285 and was statistically significant (*p* < 0.001). The ARS value was 0.627 (*p* < 0.001), while the AARS value was 0.623 (*p* < 0.001). The small difference between ARS and AARS indicates relatively stable results after adjusting the R^2^ values. The AFVIF value was 2.645, which is below the threshold of 3.3, indicating that full collinearity was not a major concern in the model. However, given the self-reported and cross-sectional nature of the data, common-method bias cannot be completely excluded. The Tenenhaus GoF value was 0.707. This value exceeds the threshold of 0.36 associated with a high value. At the same time, the SPR, RSCR, SSR, and NLBCDR indicators each had a value of 1.000. The obtained values meet the recommended criteria for these indicators (Table 8).

## 4. Discussion

The present study investigated the main factors associated with orthopedic doctors’ attitudes toward artificial intelligence and their intention to use this technology in clinical practice. The findings should be considered in the context of the increasing use of AI across different areas of orthopedic practice. The growing importance of advanced technologies in medical practice has also been highlighted in previous research conducted in other medical fields [75]. Previous studies have highlighted the potential of these technologies to support surgical procedures, medical image analysis, and clinical decision-making. Artificial intelligence has shown great intraoperative potential in real-time data processing, robotic control, and surgical strategy adaptation. AI is able to increase precision and safety, particularly during complex procedures [76]. Medical image segmentation provides crucial guidance in surgical practice, including preoperative planning and real-time navigation during operations. For instance, bone structure segmentation represents a fundamental task in AI-assisted orthopedic procedures [77]. Another significant application of AI in orthopedics is precise surgical planning, which is highly dependent on localization and anatomical landmark detection for intraoperative guidance [78]. Modern orthopedic surgery is also supported by 3D reconstruction and visualization, providing anatomical models that are important for preoperative and intraoperative guidance. A breakthrough in implementing AI within orthopedic surgery is the management of complex cases of bone cancer by combining AI models with 3D-printed replicas. In this way, individual anatomy can be analyzed, and optimal entry points for screws can be established with a high success rate [79]. In the case of clinical decision-making, surgical decision support systems play an important role in providing medical specialists with evidence-based recommendations. Decision support systems can provide personalized surgical strategies rather than relying exclusively on surgeons’ clinical experience and intuition. Their objective is to create a comprehensive framework to support medical specialists throughout the different phases of surgical procedures and to improve the precision and safety of interventions [80]. By predicting risks before the actual medical procedure, surgeons are able to make more informed decisions. One area of particular interest is spinal surgery, which is characterized by a high degree of invasiveness [81]. These previous findings are consistent with the results of the present study. The orthopedic doctors included in our research also considered medical image analysis, diagnosis, and preoperative planning among the areas in which AI could provide important benefits. Therefore, the perceived potential of AI identified in our sample reflects several of the clinical applications already described in the literature.

The research results showed that orthopedic doctors are open to the use of new emerging technologies and, in particular, artificial intelligence. A favorable perception of AI among doctors has also been identified in previous research, although limitations related to knowledge and training remain important [82]. The study showed that most respondents use digital technologies frequently or very frequently; however, only 40.3% reported using AI-based tools frequently or occasionally in medical practice. Doctors considered that AI could be used to a greater extent in the analysis and interpretation of medical images and in supporting diagnosis. This finding is consistent with previous research highlighting medical image analysis and diagnostic support as important applications of AI in orthopedics [77,78]. On the other hand, diagnosis and preoperative planning were considered the stages in which this technology could provide the most important benefits. This result is also in line with previous studies that have emphasized the role of AI in surgical planning and intraoperative guidance [78,79]. The results also showed the existence of barriers that may slow down AI adoption. Lack of knowledge and training in this field represents the main barrier identified. This is also associated with the fact that a large proportion of respondents have not participated to date in courses, conferences, or training programs on the use of AI in medicine. Moreover, doctors reported having some reservations regarding the use of this technology. The main risks perceived by them were the generation of incorrect results or recommendations, the presence of errors or biases in algorithms, and the development of excessive dependence on technology. Previous research has also shown that decisions regarding the use of new medical technologies can be influenced by several factors [83].

The results of the conceptual model showed that all the factors analyzed were positively and significantly associated with orthopedic doctors’ attitudes toward AI. Perceived usefulness had the strongest association with attitude (β = 0.27). This finding is consistent with Cicin and Çetin Gürkan [62], who examined AI acceptance among doctors and identified perceived usefulness as an important factor in doctors’ acceptance of AI. Their findings also showed the importance of other mechanisms, particularly trust and perceived ease of use. In our study, doctors who perceived AI as useful for their clinical activity also had a more favorable attitude toward its use. Digital competencies (β = 0.22) and perceived advantages (β = 0.21) were also positively associated with attitude. The importance of users’ perceptions and their interaction with digital technologies has also been highlighted in previous research conducted in the medical education context [84]. Similar findings have been reported in previous studies [85], where digital skills and the perceived benefits of technology were associated with their acceptance among healthcare professionals. Perceived ease of use and trust in AI were also significantly associated with attitude. The importance of trust in the acceptance and clinical integration of AI has also been highlighted in previous research among healthcare professionals [86,87]. These results show that doctors’ attitudes may also depend on how easy they consider AI to use and how much they trust this technology. However, previous studies have not always found positive results. Hah and Goldin [88] studied clinicians who used AI tools to support diagnosis. They found that AI did not improve clinicians’ perceived diagnostic ability. They also found a negative link between AI use and overall performance. These findings suggest that clinicians may not always have positive views of AI in clinical settings. The importance of doctors’ confidence in AI has also been highlighted in previous research examining the integration of this technology into clinical practice [89]. The five factors included in the model explained 83% of the variation in doctors’ attitudes toward AI. A strong association was also found between doctors’ attitudes and their intention to use AI (β = 0.65). Similar results have been reported in other medical populations. Shi et al. [90], in a nationwide study of 4024 doctors, found a high level of AI acceptance. Performance expectancy, social influence, facilitating conditions, and the perceived positive impact of AI were significantly associated with doctors’ intention to use AI.

In addition, doctors’ attitudes accounted for 43% of the variance in their intention to use this technology. The explanatory power of the present model can also be compared with previous PLS-SEM studies conducted among healthcare professionals. Swathi et al. [91] reported an R^2^ of 0.553 for behavioral intention to use AI among healthcare professionals in India. Stevens and Stetson [66] found that trust explained 56% of the variance in clinicians’ acceptance of a specific AI application. In comparison, the R^2^ of 0.83 obtained for attitude in our study indicates a high level of explained variance, while the R^2^ of 0.43 for intention is somewhat lower than the values reported in these studies. However, these differences should be interpreted with caution because the studies involved different medical populations, AI applications, constructs, and model specifications. A similar relationship between the evaluation of advanced medical technologies and future behavioral intention has also been reported in previous research, although from the patients’ perspective [92]. Therefore, a favorable attitude toward AI may play an important role in its future adoption in orthopedic practice. Considering these aspects, the results suggest that developing a favorable attitude toward artificial intelligence represents an important element for the future adoption of this technology in orthopedic practice. However, not all studies have reported equally positive attitudes toward AI. Heinrichs et al. [93] found that doctors still expressed concerns about AI, particularly regarding transparency, responsibility, and changes in clinical decision-making. Their results also showed that doctors with less experience and familiarity with AI tended to be more skeptical. Other findings also differ from those obtained in our study. Zheng et al. [94] found that perceived ease of use did not have a significant direct effect on clinicians’ intention to use an AI-enabled clinical decision support system. Such differences may be particularly important in orthopedics, where AI is used not only for diagnosis and decision support, but also for surgical planning, intraoperative guidance, and robotic systems. Medical specialty may also affect how doctors view AI. Huisman et al. [95] found generally positive attitudes among radiologists. However, some concerns about its impact on the profession remained. This suggests that attitudes toward AI may differ between medical specialties. This is especially relevant in orthopedics, where AI is used for diagnosis, surgical planning, and robotic procedures.

Analyzing the novelty of this study, it should be noted that it has the capacity to provide an integrated analysis of the factors that may explain the adoption of this technology among orthopedic doctors. Thus, unlike previous studies, which have often focused more on the use or benefits of AI in this field, the research conducted in this study simultaneously examined the role of perceived usefulness, perceived ease of use, trust in AI, digital competencies, and perceived advantages in shaping doctors’ attitudes toward this technology. From a practical perspective, the study provides added value because it highlights the main factors that facilitate or limit the integration of this technology into clinical practice. The results may provide useful information for healthcare facility managers and doctors regarding the factors that should be considered when integrating AI into orthopedic practice. However, greater acceptance of AI does not necessarily mean better or safer orthopedic care. A high level of trust may also become a problem if doctors rely too much on AI and overlook possible errors. For this reason, AI should be used with appropriate human oversight. External validation, clear responsibilities, and monitoring of AI use in clinical practice are also important. These aspects were not assessed in our study. Our results therefore reflect doctors’ attitudes and intentions to use AI, rather than the safety or clinical benefits of its use in orthopedic practice. Regarding the limitations of the research, it should be noted that the first limitation concerns the relatively small number of respondents who participated in this study. Thus, the limited number of respondents does not allow the results obtained to be extrapolated to the entire population under study. Another limitation identified in this research concerns the fact that the data collection process was conducted using a questionnaire that was made available to doctors through a digital platform. Thus, collecting data in this manner may lead to discrepancies between doctors’ reported perceptions and opinions and their actual behavior in practice. Another limitation concerns the cross-sectional nature of the study. Thus, the data obtained reflect doctors’ perceptions at a specific point in time, in a context in which AI-based technologies are developing rapidly. In addition, as all variables were collected from the same respondents using a self-reported questionnaire at a single point in time, common method bias cannot be completely excluded and may have contributed to the strength of some observed associations and to the high explained variance in attitude. Another limitation concerns the limited number of variables considered in the model. In addition to these, there may be other variables that, for various reasons, were not considered in this research. Another limitation concerns the sampling method, which may limit the representativeness of the sample and the generalizability of the results. In addition, the strong predominance of respondents from urban areas further limits the generalizability of the findings to orthopedic doctors at the national level. In addition, attitude and intention were measured using single items. Although these items provided a direct overall assessment of the two constructs, they may not fully capture their multidimensional nature. This may limit construct validity and should be considered when interpreting the results. Future studies could use validated multi-item scales to provide a more comprehensive assessment of attitude and behavioral intention. Another limitation is that AI was considered as a general concept in this study. We did not distinguish between different types of AI applications. Doctors may have different views of AI used for diagnosis, robotic surgery, or administrative tasks. The questionnaire did not identify the specific AI tools used by the respondents. It also did not assess their regulatory status or the frequency of use for each type of application. Therefore, self-reported AI use should be interpreted with caution. Respondents may have had different types of AI technologies in mind when answering the questions. These applications also differ in terms of risk, responsibility, and the physician’s role in the clinical decision. As no specific AI use case was provided in the questionnaire, respondents may have considered different types of AI applications when answering the questions, which limits the clinical interpretation of the reported attitudes and intentions. For this reason, acceptance may differ from one type of AI application to another. Future studies should examine these applications separately, especially in orthopedic practice. Legal liability is another issue that should be considered. In our study, 8.4% of respondents identified legal and liability issues as a barrier to AI adoption. However, we did not include legal liability as a separate variable in the model. We were therefore unable to examine whether it affected doctors’ intention to use AI. Future studies could include legal risk and professional liability in AI adoption models. This could be especially important for AI used in clinical decision-making or surgical procedures.

Based on the results obtained in this study, future studies should continue to analyze the factors that may be associated with the adoption of artificial intelligence in orthopedic practice. Thus, in the future, the conceptual model proposed in this study could be extended by considering a larger number of variables. Other variables that could be analyzed in future research include perceived risks, the cost of implementing such technology, organizational influence, and social influence. Moreover, future studies should also analyze the main differences between doctors who currently use AI in their professional practice and those who have not yet experienced its use. Future studies should also examine whether professional experience is associated with doctors’ attitudes toward this technology. Based on this information, comparative research should be conducted to identify differences in attitudes among doctors from different specialties. Furthermore, future studies should be conducted on larger samples, which would provide a better opportunity to generalize the results to the population under study.

In conclusion, this study contributes to a better understanding of the factors associated with the adoption of artificial intelligence in orthopedic practice. The results obtained may be considered a starting point for the more effective integration of AI into clinical practice.

## 5. Conclusions

In recent years, advances in technology have contributed to the growing use of artificial intelligence in medicine. Doctors have started to use AI more often for diagnosing and assessing patients and for supporting the selection of appropriate treatments. This development has been supported by the increasing digitalization of medical information. This has enabled AI-based systems to analyze large volumes of data and provide valuable information regarding the actions that doctors should take. In the field of orthopedics, AI is used extensively, particularly in the analysis of medical images. Thus, both X-rays and images obtained from CT scans provide valuable information for the diagnosis and treatment of patients. Artificial intelligence is also increasingly used in clinical decision-making. Moreover, it makes it possible to compare images with patients’ clinical data, thereby supporting orthopedic doctors in making clinical decisions. These aspects help doctors more easily determine the treatments that should be prescribed and estimate patient outcomes. In orthopedics, AI also plays an important role in surgical planning. Thus, AI-based systems have the ability to use patient data and subsequently provide information regarding the personalization of medical procedures that can be applied. Although AI currently offers a number of advantages in the field of orthopedics, these advantages must be examined together with the challenges that may arise. Thus, doctors who use this technology must remain aware that the information generated by AI-based systems depends to a large extent on the data provided to them. Therefore, the quality of the responses is influenced by the quality of the data provided. Moreover, the absence of certain information may affect the responses provided by these applications. Another issue that may arise in the use of AI in orthopedics concerns the protection and security of patient data, as well as trust in this technology. Considering the aspects presented above, it should be noted that the development of new emerging technologies and, in particular, AI is not sufficient for its integration into the field of orthopedics. Doctors’ intention to use this technology may be associated with how they perceive AI and its potential role in clinical practice. For this reason, understanding the factors associated with doctors’ attitudes toward this technology and their future intention to use it may provide relevant information regarding its potential use in practice. In this study, these relationships were examined among a sample of orthopedic doctors practicing in Romania who were recruited using snowball sampling. However, the findings reflect doctors’ perceptions and intentions rather than actual AI use. The perceived benefits reported in this study should not be interpreted as evidence of improved clinical outcomes or patient care.

## Figures and Tables

**Table 1:** Reliability analysis of the constructs included in the conceptual model.

Construct	Cronbach’s α	Composite Reliability	AVE
Perceived Usefulness (PU)	0.912	0.934	0.740
Perceived Ease of Use (PEOU)	0.894	0.922	0.704
Trust in AI (TRUST)	0.913	0.935	0.743
Digital Competencies (DIGCOMP)	0.877	0.911	0.671
Perceived Advantages (PADV)	0.902	0.927	0.719

**Table 2:** Indicator loadings and cross-loadings of the measurement model.

Indicator	PU	PEOU	TRUST	DIGCOMP	PADV	ATT	INT
Q15_1	0.858	−0.244	−0.048	−0.186	−0.147	0.359	−0.089
Q15_2	0.827	0.366	−0.100	0.144	0.197	−0.362	0.099
Q15_3	0.878	−0.196	0.025	0.007	−0.117	−0.058	−0.012
Q15_4	0.865	−0.049	0.028	0.151	−0.213	−0.318	0.048
Q15_5	0.869	0.139	0.090	−0.110	0.288	0.153	−0.042
Q16_1	0.075	0.787	−0.128	−0.112	−0.221	0.357	0.012
Q16_2	0.425	0.811	−0.029	−0.117	−0.015	−0.316	0.083
Q16_3	0.333	0.854	0.061	0.066	0.132	−0.345	0.101
Q16_4	−0.319	0.892	0.034	0.119	0.111	0.270	−0.045
Q16_5	−0.477	0.846	0.050	0.025	−0.030	0.339	−0.145
Q17_1	0.338	−0.180	0.857	0.157	−0.412	0.147	−0.025
Q17_2	−0.153	0.049	0.876	−0.006	−0.002	0.157	−0.163
Q17_3	−0.435	0.051	0.865	−0.080	0.418	0.020	0.040
Q17_4	0.393	−0.144	0.836	−0.064	−0.161	−0.029	0.011
Q17_5	−0.124	0.214	0.875	−0.007	0.146	−0.293	0.137
Q18_1	−0.402	0.069	−0.020	0.844	0.370	0.362	0.139
Q18_2	−0.008	−0.264	−0.169	0.827	−0.007	0.329	0.323
Q18_3	0.399	−0.077	−0.082	0.867	−0.255	−0.071	0.263
Q18_4	0.071	0.167	0.156	0.797	−0.076	−0.298	−0.389
Q18_5	0.144	0.124	0.136	0.758	−0.256	−0.275	−0.399
Q19_1	−0.221	−0.231	−0.019	−0.167	0.864	0.387	−0.102
Q19_2	0.094	0.385	−0.073	0.137	0.819	−0.302	0.094
Q19_3	0.331	−0.257	−0.052	−0.021	0.830	0.000	−0.045
Q19_4	0.329	−0.038	0.038	0.157	0.856	−0.298	0.074
Q19_5	−0.310	0.150	0.100	−0.099	0.868	0.166	−0.018
Q20	0.000	0.000	0.000	0.000	0.000	1.000	0.000
Q21	0.000	0.000	0.000	0.000	0.000	0.000	1.000

**Table 3:** Inner VIF Values of the Predictor Constructs.

Endogenous Construct	Predictor	Inner VIF
ATT	PU	2.83
ATT	PEOU	2.05
ATT	TRUST	1.38
ATT	DIGCOMP	1.93
ATT	PADV	2.76
INT	ATT	1.00

**Table 4:** Discriminant validity assessment using the Fornell–Larcker criterion.

Construct	PU	PEOU	TRUST	DIGCOMP	PADV	ATT	INT
PU	0.860	0.642	0.493	0.640	0.750	0.742	0.539
PEOU	0.642	0.839	0.443	0.563	0.659	0.669	0.474
TRUST	0.493	0.443	0.862	0.402	0.419	0.525	0.587
DIGCOMP	0.640	0.563	0.402	0.819	0.634	0.620	0.535
PADV	0.750	0.659	0.419	0.634	0.848	0.721	0.496
ATT	0.742	0.669	0.525	0.620	0.721	1.000	0.610
INT	0.539	0.474	0.587	0.535	0.496	0.610	1.000

**Table 5:** KMO measure and Bartlett’s test of sphericity.

Indicator	Value
Kaiser–Meyer–Olkin (KMO) Measure of Sampling Adequacy	0.823
Bartlett’s Test of Sphericity—Approx. Chi-Square	5305.990
df	300
*p*-value	<0.001

**Table 6:** Results of hypothesis testing.

Hypothesis	Relationship	β	*p*-Value	Decision
H1	Perceived usefulness → Attitude	0.27	<0.01	Supported
H2	Perceived ease of use → Attitude	0.18	<0.01	Supported
H3	Trust in AI → Attitude	0.18	<0.01	Supported
H4	Digital competencies → Attitude	0.22	<0.01	Supported
H5	Perceived advantages → Attitude	0.21	<0.01	Supported
H6	Attitude → Intention to adopt AI	0.65	<0.01	Supported

**Table 7:** Coefficient of determination (R^2^).

Endogenous Construct	R^2^
Attitude toward AI use	0.83
Intention to adopt AI	0.43

**Table 8:** Model quality indicators.

Indicator	Obtained Value	Validation Criterion
Average path coefficient (APC)	0.285, *p* < 0.001	*p* < 0.05
Average R-squared (ARS)	0.627, *p* < 0.001	*p* < 0.05
Average adjusted R-squared (AARS)	0.623, *p* < 0.001	*p* < 0.05
Average full collinearity VIF (AFVIF)	2.645	Acceptable ≤ 5; ideally ≤ 3.3
Tenenhaus GoF (GoF)	0.707	Small ≥ 0.10; medium ≥ 0.25; large ≥ 0.36
Simpson’s paradox ratio (SPR)	1.000	Acceptable ≥ 0.70; ideally = 1
R-squared contribution ratio (RSCR)	1.000	Acceptable ≥ 0.90; ideally = 1
Statistical suppression ratio (SSR)	1.000	Acceptable ≥ 0.70
Nonlinear bivariate causality direction ratio (NLBCDR)	1.000	Acceptable ≥ 0.70

## Data Availability

The original contributions presented in this study are included in the article. Further inquiries can be directed to the corresponding authors because the study data were collected within an institutional setting and are subject to ethical and data protection restrictions.
